# Perspective: bidirectional exosomal transport between cancer stem cells and their fibroblast-rich microenvironment during metastasis formation

**DOI:** 10.1038/s41523-018-0071-9

**Published:** 2018-07-16

**Authors:** Gábor Valcz, Edit Irén Buzás, Zoltán Szállási, Alexandra Kalmár, Tibor Krenács, Zsolt Tulassay, Péter Igaz, Béla Molnár

**Affiliations:** 1 Molecular Medicine Research Unit, Hungarian Academy of Sciences, Budapest, Hungary; 2 2nd Department of Medicine, Semmelweis University, Budapest, Hungary; 3 MTA-SE Immuno-Proteogenomics Extracellular Vesicle Research Group, Hungarian Academy of Sciences, Budapest, Hungary; 4 Department of Genetics, Cell- and Immunobiology, Semmelweis University, Budapest, Hungary; 5 Computational Health Informatics Program (CHIP), Boston Children’s Hospital, Harvard Medical School, Boston, USA; 6 1st Department of Pathology and Experimental Cancer Research, Semmelweis University, Budapest, Hungary

## Abstract

Carcinomas are complex structures composed of hierarchically organized distinct cell populations such as cancer stem cells and non-stem (bulk) cancer cells. Their genetic/epigenetic makeup and the dynamic interplay between the malignant cell populations and their stromal fibroblasts are important determinants of metastatic tumor invasion. Important mediators of these interactions are the small, membrane-enclosed extracellular vesicles, in particular exosomes. Both cancer cell and fibroblast-derived exosomes carry a set of regulatory molecules, including proteins and different species of RNA, which cooperatively support metastatic tumor spread. Here, we briefly overview potential links between cancer stem cells and the exosome-mediated fibroblast-enriched metastatic niche formation to discuss their role in the promotion of tumor growth and metastatic expansion in breast carcinoma models.

## Introduction

Metastatic tumor progression, a stepwise sequence of events including local invasion, intravasation, survival in the circulation, extravasation, and colonization, is responsible for 90% of cancer-associated mortality.^1,2^ In this process, cancer cells with the capacity of tumor initiation and repopulation, i.e., cancer stem cells (CSCs), break away from the primary tumor and colonize the same or different organs (i.e., they form local or distant metastasis).^3^ In recent years, metastatic tumor spreading has been viewed as a process that involves a dynamic interplay between cancer cells and their non-malignant microenvironment. Based on this, the success of metastasis formation depends not only on genetic/epigenetic deregulation of cancer cells that ensures survival advantage (analogous to Darwinian evolution), but also on the support of the tumor adjacent stromal microenvironment, frequently called “niche”.^4,5^ Soluble and vesicular regulators from CSC and non-stem-like (i.e., bulk) cancer cells can influence the niche in several ways including modulation of angiogenesis and exert a broad range of effects by which they perturb functions of the immune system.^3,6^ Furthermore, tumor-secreted regulators transform normal stromal cells into cancer-associated fibroblasts (CAFs), which may support cancer cells, including the development of stem-like properties and therapy resistance.^7–9^

## Stem cells, bulk cells, and their niche

Solid tumors harbor a cellular complexity that exhibits hierarchical organization and functional heterogeneity, which is also reflected by the distinct proliferative and differentiation capacities of the cells. The classical concept of CSC (or hierarchical) theory states that a small subpopulation of tumor cells, that are widely considered to arise from normal stem cells, show long-term self-renewal potential and the ability of tumor initiation and lineage transition.^10–12^ CSCs show upregulated signaling pathways essential in stem cell biology, such as Notch, Wnt, and Hedgehog.^13^ They acquire epigenetic and genetic changes required for tumorigenicity, and they are capable of repopulating the tumor after radiotherapy or chemotherapy.^11,14^ CSCs generally identified with detection of specific stem cell markers. In breast cancer, CSCs are frequently described as a CD44^+^/CD24^-/low^/Lineage^−^ (mammary epithelial lineage marker negative) or/and an ALDH^+^ subpopulation.^15–17^ Expression of the cell-surface glycoprotein CD133, an accepted CSC marker and a prognostic factor in breast cancer, was positively associated with aggressive tumorigenicity showing vasculogenic mimicry (i.e., cancer cells gain endothelial phenotype and form vessel-like networks) and hormone therapy (HT) resistance.^18,19^ An interesting question is the relative appearance of CD44^+^/CD24^-/low^ and CD133 expression pattern in the given CSC cell. For example MDA-MB-231 culture contains >94% CD44^+^/CD24^−/low^ and ~26% CD133^+^ cells which suggests only a partial overlap between CSC markers.^18^ In contrast, Wright et al. found no overlap between these phenotypes in BRCA1 deregulated tumors, and they suggest two distinct CSC populations.^20^ Populations with no overlap with CSC marker expression (i.e., CD133^low^/CD44^high^ and CD133^high^/CD44^low^) equally display stem-like and partially different features, such as HT resistance in case of CD133^high^ cells.^19^ Activation of leptin receptor (a non-exclusive breast cancer CSC marker)-induced pathways (e.g., NANOG, PI3K/AKT, MEK1, and JAK2-STAT3) has also been shown to be required for the induction and the maintenance of stem-like properties.^21,22^

**Fig. 1:**
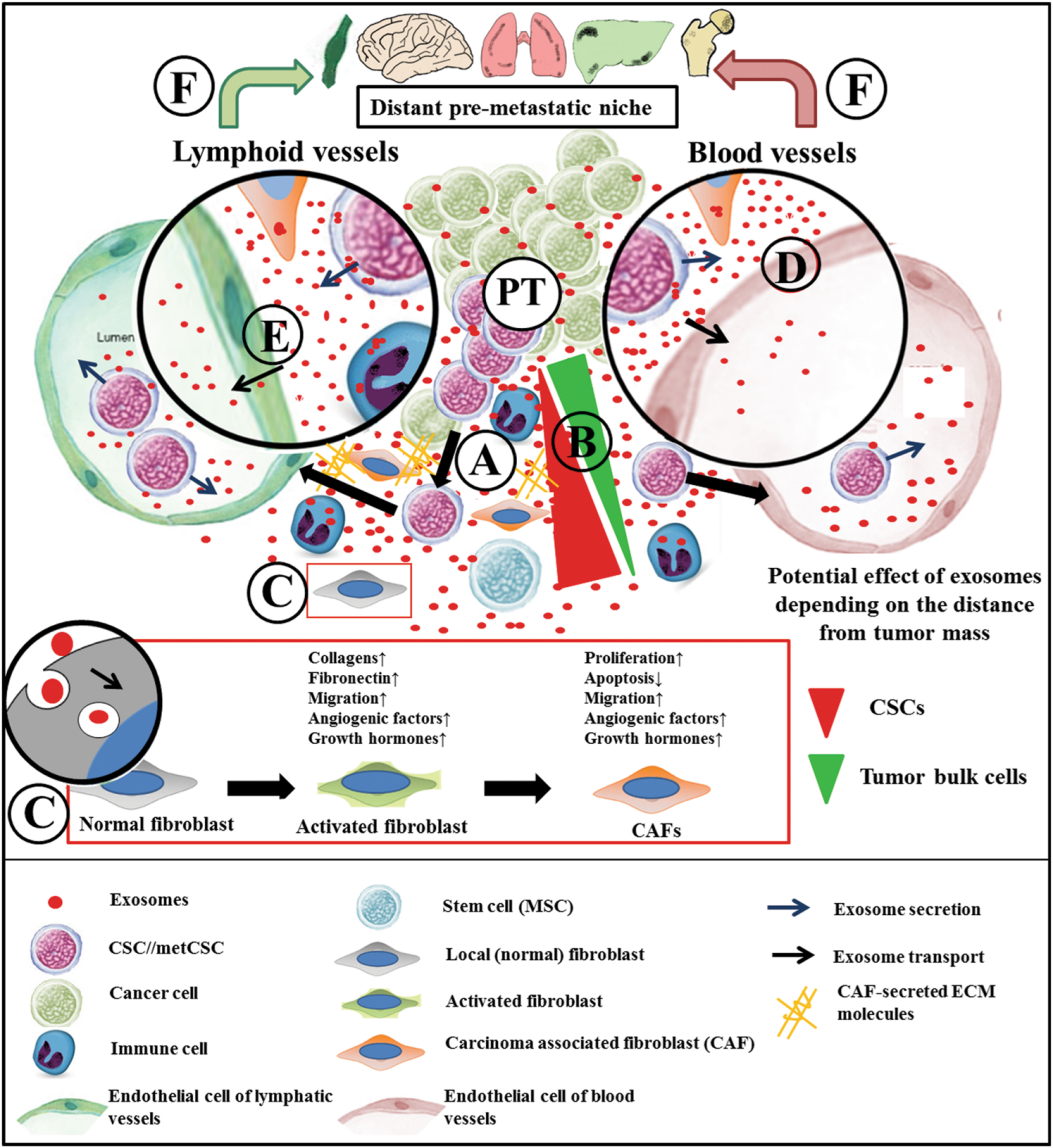
Exosomes of disseminated CSCs (**a**) with bulk cells of a primary tumor (PT) cooperating in the formation of a local metastatic niche near the tumor mass. We speculate that during further migration of tumor cells, the effects of migrating CSC-derived exosomes may have more impact than of exosomes secreted by the distant tumor mass (**b**). Induction of CAF differentiation is probably a multistep process (i.e., NF-activated fibroblast-CAF sequence). It is accompanied by the appearance of the tumor promoting effects of CAFs (**c**). CSC-derived exosomes are detectable both in the lymphatics and in blood circulation. They may originate from the stroma and may either annihilate the endothelial tight junctions (**d**) or they undergo an active transport by the endothelial cells (**e**). Evidently, these exosomes may also originate from circulating CSCs and may play a role in the formation of pro-metastatic site in distant organs (**f**)

CSCs derived from the primary tumor mass (primary CSC) generate transit-amplifying progenitors and their short-lived derivatives (i.e., clones of bulk cells) with phenotypic and functional heterogeneity, but without tumor-initiating capacity.^15,23^ Individual CD44^+^/CD24^–/low^ stem-like cells are detectable in the tumor-invasive edge adjacent to the tumor stroma (Fig. 1a). Their expression profile seems to be different from that of ALDH^+^ (epithelial-like) CSCs, with the latter usually localized in the internal zones of breast primary tumors. However, the transition between these two CSC phenotypes has been observed, suggesting plasticity between CD44^+^/CD24^–/low^ cells of metastatic capacity referred here as metastatic (met)CSCs and those of primary CSCs.^24^ Upon detachment from tumor nests, cancer cells partially lose their epithelial phenotype and acquire mesenchymal and stem cell characteristics (epithelial-to-mesenchymal transition (EMT)).^25^ Cancer cell detachment without metastasis initialization is thought to be a relatively frequent event, but most of these cells are either eliminated by an effective immune surveillance mechanism or lack the ability to form a new tumor.^1,26^ While the metastatic potential is considered to be a CSC-specific property, it still largely depends on the microenvironment.^3,27^ The relationship between CSCs and their niche appears to be bidirectional: cancer cells can modify their microenvironment, and conversely, according to the Paget’s seed and soil hypothesis, the niche as a “fertile soil” specifically enables both self-renewal of CSCs and produce all other carcinoma cells of the tumor mass.^3,28,29^ This niche can be defined as a supportive and receptive tissue microenvironment undergoing a series of molecular and cellular changes to form metastatic sites.^30^ The evolution of this extrinsic regulatory system is a multistage process which can be divided into (i) niche construction, (ii) expansion, and (iii) maturation.^28^ In niche construction, the activated recipient cells comprising of epithelial, immune, fibroblast-like cells, and extracellular matrix (ECM) components may improve cancer cell survival before the arrival of metCSC by generating a hospitable microenvironment. This process is mediated by cell–cell interactions, soluble factors, and exosomal signaling (see below).^28,30,31^ Pre-metastatic niche is conducive of metCSCs by controlling their homing to the metastatic sites.^3,6,11,28^ After metCSCs integration into the metastatic niche, paracrine communication will be dominant (Fig. 1b), which promote tumor malignancy during the niche expansion and maturation.^3,28^

## Fibroblasts in cancer niche

Niche components have been shown to contribute to tumor progression by regulating the homing, anchoring, self-renewal potential, and the dedifferentiation of CSCs. CAFs as main stromal components of solid tumors can differentiate from several cell types including local, resting, normal fibroblasts (NFs) and mesenchymal stem cells (MSCs).^32,33^ These precursors may acquire a transitional, activated (α-SMA and vimentin positive) phenotype, e.g., with increased secretion of collagens and fibronectin (characteristic of activated fibroblasts) which may provide structural support and anchorage to metastasizing cancer cells.^7,33,34^ The precursors gain a pronounced secretory phenotype with CAF-specific gene expression pattern as they finally transform to CAFs (e.g., fibroblast-activated fibroblast-CAF sequence; Fig. 1c). NFs and CAFs can be distinguished by their differential epigenetic features,^35^ miRNA patterns,^36,37^ and expression of NF-related molecules, namely, reduced levels of TIMPs and p85α proteins.^9,38^ CAFs play important roles in reprogramming of the tumor microenvironment through (i) maintenance of the reactive stroma, e.g., by secreting TGFβ1 and PDGF; (ii) induction of angiogenesis, e.g., by producing VEGF, SDF-1, and FGF2; (iii) induction of tumor cell proliferation, e.g., by SDF-1, IGF2, and Gremlin-1 production; and (iv) facilitating tumor invasion, e.g., by producing TGFβ1, HGF, MMPs, and tenascin.^33,39^ They also secrete large amounts of ECM molecules including collagen, laminin, and fibronectin. In addition, the modulated ECM can serve as a reservoir for oncogenic signals,^40–42^ which may influence CSC properties including migration and drug resistance.^43–45^ Breast CAFs isolated from HER2-positive, triple-negative, and ER-positive breast tumors showed distinct gene expression patterns and functions.^46^ This study showed that properties of CAFs are highly influenced by the adjacent cancer cells and it also supports the tumor-stroma co-evolution hypothesis, which suggests that CAFs can fine tune their supporting role to the specific tumor cells.^46^

## Exosomes in the breast cancer niche

Beside soluble regulators, the complex interaction between niche cells and the tumor epithelium also involves extracellular vesicles (EVs), such as microvesicles (ectosomes) and exosomes. Exosomes are small (~50–150 nm in diameter), multivesicular body (MVB)-derived EVs secreted into the extracellular space, which play important role in the maintenance of homeostasis of the releasing cells.^47–49^ As potent intercellular communicators, they carry specific molecules such as major histocompatibility complex, MVB proteins (e.g., ALIX and TSG101), tetraspanins (e.g., CD63, CD81, and CD82), and chaperones including heat shock proteins (Hsp60, Hsp70, and Hsp90).^47,50^ They also contain proteins, as well as coding and non-coding RNAs.^51–53^ Exosomal DNA can trigger cytosolic receptors (e.g., AIM2) of immune cells and result in tumor supporting inflammatory cytokine secretion.^54^ Horizontal transfer of genomic (g)DNA to the nucleus of recipient cell is possible via EVs (with ~30–1000 nm in diameter), and this gDNA-coded mRNA and related functional proteins can also be expressed.^55^ Based on the available data the transformation potential of the exosome-like EV’s gDNA is temporary and limited to uptake, as well as morphological changes without genomic integration in immortalized fibroblast cells.^56^

Endocrine and paracrine or autocrine exosomal communication may be (i) a receptor-mediated event, (ii) a result of fusion, or (iii) endocytosis with subsequent modification of the protein expression in the recipient cells.^57,58^ Exosomes from a primary tumor can transit to the lymphatics or/and to the blood circulation (Fig. 1d, e), and they can also reach cells of distant organs (Fig. 1f).^59–61^ Importantly, this endocrine effect shows organ specificity (i.e., integrin dependency), which involves activation of recipient stromal cells during niche construction before the arrival of the metastatic cancer cells.^31^ Circulating tumor-derived exosomes may carry the substrates of tumor-specific mutations, cell-specific proteins, and RNAs including micro(mi)RNAs.^62^ Monitoring changes in circulating exosomal proteins (e.g., CD24 and survivin-2B) and miRNAs (e.g., miR-21 and miR-1246) is a promising approach, which may support future early diagnosis and staging of breast cancers from patients’ sera in liquid biopsy.^63,64^ Generally, the endocrine and paracrine exosomal effects result in the formation of tumor-supporting microenvironment. This also includes a modulated immune environment with altered recruitment of immune cells, altered presentation of tumor antigens, downregulated immune activation, and increased immunosuppression,^65^ as well as a pronounced CAF differentiation.

## Effect of cancer cell-derived exosomes on CAFs and their precursors

Both activated fibroblasts and fully differentiated CAFs can influence cancer initiation and progression.^7,40^ These cells probably appear early in niche construction and will be prominent stromal elements in the later phases of metastatic niche formation.

Exosomal proteins can be more effective in the conversion of MSCs and NFs to activated fibroblast or a CAF-like phenotypes compared to their soluble counterparts. The best example for this is TGF-β bound to exosomal heparane sulfate proteoglycan. In the prostate cancer secretome, extracellular TGF-β associated with exosomes is found in smaller amounts than soluble TGF-β, 20% vs. 80%.^66,67^ However, of identical amounts of TGF-β the exosome membrane-bound ligand has significantly more pronounced effect than the unbound molecule.^66^ Exosomal TGF-β of different tumors including breast cancer cause increased expression of TGF-β receptors (TGF-βRI and II) and activation of SMAD-dependent and SMAD-independent (e.g., PI3K/AKT) pathways.^66,68,69^ Furthermore, exosomal, but not soluble TGF-β leads to differentiation of a biochemically distinct activated fibroblast/CAF-like phenotype.^70^ NFs or MSCs exposed to tumor cell-derived exosomal TGF-β induced angiogenesis (via uPA, HGF, VEGF-A, FGF2 secretion) in co-cultured endothelial cells. Also, they caused enhanced migration/invasion (i.e., by secretion of MMP1, MMP3, and MMP13), expansion, and proliferation of tumor cells.^66,70^

**Fig. 2:**
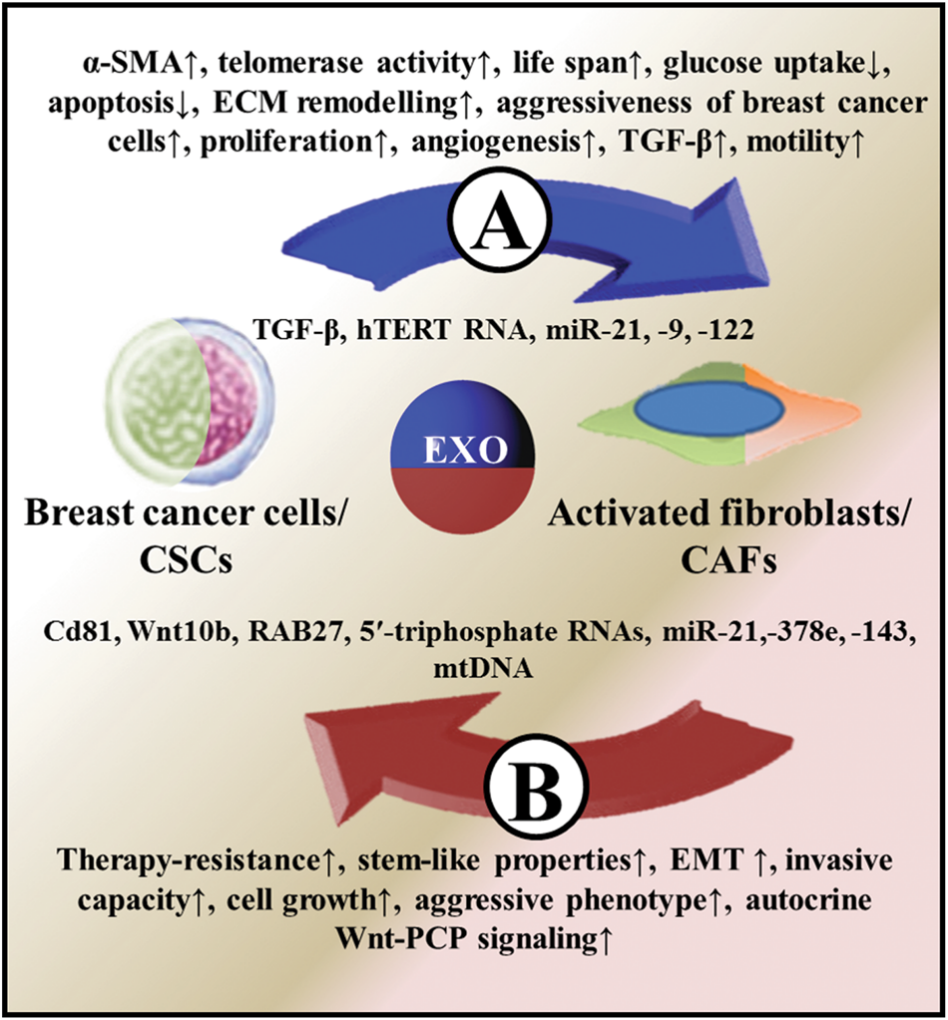
Exosomal crosstalk between breast cancer cells and activated fibroblasts/CAFs. Breast cancer cell-derived exosomal molecules and their effect on activated fibroblasts/CAFs are illustrated in (**a**). (**b**) shows the effect of activated fibroblast/CAF-derived exosomes on breast cancer cells/CSCs

Breast cancer-derived exosomes also carry different RNA species and are capable of modifying the properties of recipient-activated fibroblast/CAF precursors in the tumor microenvironment. As an important example, exosomes from different tumor cells including MCF-7 breast cancer line, contain human telomerase reverse transcriptase (hTERT) mRNA. Exosomal hTERT mRNA can be translated into a fully active telomerase enzyme that can induce increased proliferation in recipient (telomerase-negative) fibroblasts. Furthermore, the transferred hTERT mRNA also protected the cells from replicative senescence and DNA damage.^71^ Beside mRNAs, exosomal miRNAs are seen as the most important regulators of the tumor microenvironment. miRNAs are secreted aberrantly in many types of cancer and regulate gene expression through destabilization, degradation, and translational inhibition of mRNAs.^62^ Furthermore, certain microRNAs (e.g., miR-21 and 29a) can activate pattern-recognition receptors such as Toll-like receptors TLR3 and TLR8, and promote a pro-metastatic inflammatory microenvironment.^72,73^ miR-21 is known as a critical regulator of both fibroblast activation and CAF formation.^74–76^ In agreement with this, miR-21 induces NF activation (i.e., increased α-SMA, FAP, and SDF-1 expression) and promotes the proliferation and invasion of the stromal cells in breast phyllodes tumors.^77^ miR-21 suppresses *TIMP3* in NFs^78^ which is sufficient for the acquisition of CAF phenotype.^9^ Furthermore, miR-21 also influences TGF-β-induced fibroblast activation through several pathways, e.g., by the suppression of *PDCD4*, an inhibitor of α-SMA and VEGFA^79^ or by binding to *Smad7* mRNA which is a suppressor of TGFβRI-II/Smad2/3 pathway-directed CAF formation.^75,77^ Other miRNAs, such as miR-9, can modify the signature of genes correlating with cell motility and ECM organization (i.e., EFEMP1, COL1A1, and MMP1) in breast NFs.^80^ Furthermore, miR-122 can reprogram energy metabolism of stromal fibroblasts via suppressing their glucose uptake, which promotes pre-metastatic niche partially by increased glucose availability of cancer cells.^81,82^ These changes (as presented in Fig. 2a) are associated with rapid progression, poor overall survival, and secondary metastasis in breast cancer.

An important question is whether there are any differences between bulk cells and tumor-initiating CSCs in their exosomal content and effect on CAF precursors. In prostate adenocarcinoma, exosomes separated from the CSC and bulk tumor cell fractions showed clearly different effects on CAF precursors, which is less studied in breast cancer.^83^ Some breast cancer studies used purified exosomes from MDA-MB231 cultures with predominant CD44^+^/CD24^–/low^ population.^84^ However, merely being CD44^+^/CD24^–/low^ does not meet all criteria of CSCs, and it may indicate only some degree of stem-like properties. Cho et al. compared the effect of exosomes secreted by basal (MDA-MB231) and luminal (MCF-7) breast cancer lines on MSCs. Importantly, exosomes from the two cell lines could stimulate different signaling pathways associated with the fibroblast-like transformation of MSCs.^69^ However, how this difference can be related to the diverse receptor expression and signaling of these cell lines and to their different tumor stem/progenitor cell content needs further clarification.

## Effect of CAFs exosomes on breast cancer cells and CSCs

Exosomal traffic from fibroblastic cells induces clinically important properties of cancer cells including invasive capacity, stem-like properties, and therapy resistance. Here, we discuss the effect of CAF exosomal proteins, DNA, and different RNA species on breast cancer cells.

CD81 on exosomes derived from mouse fibroblasts and patient-derived CAFs can stimulate protrusive activity and motility of cancer cells by mobilizing autocrine Wnt-PCP pathway.^85^ CAF-like (i.e., p85α^−/−^) fibroblast-derived exosomes can also activate the Wnt pathway by delivering Wnt10b protein resulting in increased migration, EMT, and cytoskeletal remodeling of breast cancer cells.^38^

Exosomal RNAs can influence treatment response of breast cancer cells via activation of interferon-related DNA damage resistance signature. Mirjam et al. described that paracrine exosomal 5′-triphosphate (5′ppp) RNAs activate the pattern recognition receptor RIG-I in the cytoplasm of cells.^86^ In agreement with this, fibroblast-derived exosomal 5′ppp RN7SL1 RNA, as danger-associated molecular pattern, can activate RIG-I.^87^ This pathway cooperates and converges with juxtacrine pathways such as STAT, which facilitates the transcriptional response to NOTCH and expand therapy-resistant tumor initiating breast cancer cells.^86,87^ Primary CAF-derived exosomal miRNAs, such as miR-21, miR-143, and miR-378e promote anchorage-independent cell growth and EMT phenotype in breast cancer cells. Furthermore, these miRNAs also induced a de-differentiation process toward stem-like state with increased expression of Oct3/4, Nanog, and Sox2 markers and the aggressiveness of breast cancer cells.^88^

The mitochondrial (mt)DNA levels and mutational status within cancers are associated with the development of resistance to therapies. CAF-derived mtDNA^high^ exosome from HT-resistant breast cancer patients treated metabolically dormant populations and HT-naive breast cancer cells promoted an escape from metabolic quiescence and developed HT-resistant disease.^89^

Several of the above-described experiments (Fig. 2b) used culture with vastly different CD44^+^/CD24^-/low^ cell rates, in which CAF-derived exosomal regulators supported metastatic and/or stem cell-like properties. This phenomenon partially overlaps with the “extrinsic CSC theory” suggesting that all cancer cells are functionally equivalent, but they display heterogeneous behavior as a function of extrinsic cues.^90^ The hypothesis that cancer cells gain CSC characteristics exclusively by the effect of external signals should be handled with caution. The MDA-MB231 experiments^38,85,86^ support the relevance of this theory when a cell population with stem-like properties is used. In this case, among external signals CAF-secreted exosomes can modify the properties of these cells toward a definite metastatic and tumor-initiating phenotype. Assuming that not only the acquisition of metastatic potential but also the metCSC to CSC transformation take place in the primary tumor, the extrinsic CSC theory can be brought into line with the “dynamic heterogeneity” metastatic model. According to the latter theory, metCSC subpopulations are generated at a high rate in a primary tumor; however, these variants are relatively unstable.^91^ In our case, this model could be used with the exception that these dynamic processes are at least partly directed by fibroblast-derived exosomal signals.

## Clinical perspectives and future directions

As we discussed here, exosomes have significant pathological relevance in breast cancer progression. Inhibition of exosomal communication between cancer cells and the stromal microenvironment may have therapeutic potential.^92,93^ GW4869 is a neutral sphingomyelinase inhibitor that blocks ceramide-mediated inward budding of MVBs and exosome release; furthermore, it is widely used in breast cancer cell and CAF experiments.^38,92,94^ In combination with chemotherapeutic agents, it significantly reduced the survival of carcinoma cells in co-cultures and mouse experiments.^95,96^

In principle, CSCs could be eliminated by using exosomes with modified surface (to facilitate targeted uptake) and cargo (delivering drugs, peptides, proteins, or RNAs with low toxicity and immunogenicity).^97^ Exosomes produced by immature dendritic cells and loaded with doxorubicin have integrin-dependent anti-tumor effect to MDA-MB231 cells in a mouse model.^98^ Not only immature dendritic cells-derived, engineered exosomes, but also reprogrammed cancer cell-derived or naive bone marrow-derived MSC exosomes may have a therapeutic effect or may delay cancer recurrence. Transfected breast cancer cell-derived, miR-134 carrying exosomes can reduce target protein expression (i.e., STAT5B and Hsp90), cell migration, invasion, and enhance anti-Hsp90 drug (cisplatin)-induced apoptosis in recipient cells.^99^ Furthermore, uptake of GE11 (an epidermal growth factor receptor (EGFR) ligand) surface protein-enriched, and let-7miRNA tumor suppressor containing HCC70 cell-derived exosomes can inhibit EGFR-positive breast tumor development in vivo.^100^ MSC-derived, cell cycle inhibitory miRNA containing exosomes increased the number of CD44^−^ cells in CD44^+^/CD24^–/low^ (MDA-MB231) culture suggesting that these exosomes play a crucial role in the dormant state of breast CSCs.^101^ However, although these experiments are still in pre-clinical phase, the promising results suggest their possible importance in the future of cancer therapies.

In conclusion, the complex exosomal crosstalk between breast CSCs and fibroblastic cells plays key roles from the early steps of niche formation, through metastatic growth, to further metastasis initiation. The prominent role of exosomal crosstalk in the metastatic cascade is well justified by the effect of CSC-derived and particularly metCSC-derived exosomes on non-malignant cells. Exosomal interactions are also important in defining steps of CAF differentiation and regulating precursor cell functions in niche construction. Furthermore, exosomal signal delivery can also be important in CAF heterogeneity possibly attributed to tumor-stroma co-evolution and potential paracrine communication. Therefore, using engineered, specific exosomes against CSCs, or blocking cancer–stroma interactions hopefully will be of great significance for cancer therapies.
